# I Want to be Known as a Whole Human Being: A Qualitative Study About Patients’ Experiences of Empathy in Health Care

**DOI:** 10.1177/23743735251369702

**Published:** 2025-08-21

**Authors:** Johanna von Knorring, Arja Lehti, Kristina Lindvall, Olof Semb

**Affiliations:** 1 Department of Clinical Sciences, Professional Development, 367314 Umeå University, Umeå, Sweden; 2 Department of Epidemiology and Global Health, 174480 Umeå University, Umeå, Sweden

**Keywords:** empathy, professionalism, physician–patient relationship, communication, medical education, grounded theory

## Abstract

Empathy is crucial in forming a good relationship between patients and healthcare providers. Research on empathy in health care has largely focused on healthcare providers and students and less on the patient's perspective. This study's aim was to investigate patients’ experience of empathy in healthcare interactions, to better understand and accommodate to patients’ needs. Semi-structured interviews with patients were analyzed inspired by constructivist Grounded Theory. Three categories were formed—“I need you to focus on me,” “I am more than my disease” and “I know myself and the disease” and the core category: “I want to be known as a whole human being.” This study has provided a deeper insight on how empathy can be operationalized in healthcare interactions from a patient perspective. Patients experience health care interactions as dependent on empathic engagement and hampered by inequity due to knowledge hierarchies and lack of personal sharing. This study emphasizes patientś need for doctors to be more personal, and to experience a mutual sharing with their doctors to not feel vulnerable or left out in the consultation.

## Introduction

The ability to empathize is fundamental to understanding the feelings and actions of others, and to share the affective states of people in our surroundings.^1^ Although a frequently studied phenomenon, there is a lack of consensus regarding the definition of empathy^2^ and similar concepts such as compassion.^3^ Further, these concepts are expressed and perceived differently depending on cultural settings and therefore important to investigate in different contexts.^4,5^

In medical context, empathy is often defined as the ability to understand and acknowledge the feelings of another, resulting in a helpful response from the observer. There has been a tendency among medical scholars to distinguish between two types of empathy: cognitive and affective empathy.^6^ Cognitive empathy focuses on the understanding of the patient's experiences, relying on intellectual cues and responses.^7^ Affective empathy is the notion that emotional engagement is included and necessary to fully understand another's situation.^8^ The dichotomy between cognitive and affective empathy has led to unnecessary confusion and in a recent review article,^9^ researchers have proposed a more all-encompassing definition of empathy in the medical context drawing on concepts such as engaged curiosity.^8^The concept of engaged curiosity elaborated by Halpern encourage doctors to not only be aware of but also interested in, both the emotions of the patients as well as their own personal emotional response while interacting with patients.^8^

Despite the conceptual ambiguity, empathy has proven to be an essential component in the relationship between patients and healthcare providers^10^ as it has been shown to correlate to patient satisfaction and compliance,^11,12^ as well as positive clinical outcomes such as faster recovery from common cold and alleviate suffering in pain-related conditions.^13,14,15^ Further, the importance of empathy and compassion in health care is stressed in the Francis report where a perceived lack of these qualities is connected to negative patient outcomes.^16^

Research on empathy in health care has largely focused on healthcare providers and students^17^ and less on the patient's perspective.^3,18^ A recent study concludes that empathy has a mediating role in communication thus rendering greater patients’ satisfaction.^19^ A systematic review from 2009, identified empathy or effects of empathy to be crucial in the forming of good relationships between patients and healthcare providers.^20^ In a recent study patients, as well as doctors, struggle with the concepts of sympathy, empathy and compassion but concluded that both empathy and compassion was crucial to alleviate patient suffering.^21^

In today's health care, where a person-centered care is promoted as ideal,^22^ it would be valuable to focus even more on the recipients of empathy in clinical practice and how they experience empathy in interactions with health care. In this study we aim to investigate patients’ experience of empathy in healthcare interactions, striving to move beyond conceptual descriptions of empathy. By doing so we aim to reach a more in-depth understanding of how patients perceive doctors as empathic, or lacking in empathy, to better understand and accommodate to patients’ needs.

## Method

### Participants

The sampling for this study was purposive.^23^ A patients’ organization in northern Sweden was approached to relay information and a request of participation to people with varying healthcare experiences. Informants were also recruited directly through an outpatient clinic. The inclusion criteria were as follows: age 18 years or older and must have had one or more healthcare visits during the past 12 months. Those who contacted the researchers and met the criteria were contacted with information about the study and the possibility to withdraw from the study at any time and without explanation. They also received verbal briefs on the topic of the interview and were asked to choose a time and a place for the interview. All participants signed a standardized informed consent before the interview. This study was granted ethical approval for research regarding humans from the regional ethics committee (Dnr: 2016/50-31).

In total, 18 informants (11 from an outpatient clinic and 7 through the patients’ organization) chose to participate. They ranged between 25 and 90 years of age, and 11 identified as female while seven identified as male. The participants had interacted with healthcare personnel—doctors and/or nurses—between 1 and 16 times during the past 12 months. Some of the participants were chronically ill, some had multiple diseases, and some had sought help for a single temporary illness or injury. No previous relation existed between the interviewers and the participants prior to the study.

### Data Collection and Documentation

The semi-structured interview guide comprised open-ended questions to encourage the informants to speak freely and in their own words, to obtain rich content. Examples of questions were: How would you describe empathy? Can you tell me about an empathic encounter in health care? Using the interview guide as a framework, the interviewers strived to be mindful and flexible in asking follow-up and prompting questions when necessary.

Data was collected between February 2019 and October 2020; due to the COVID-19 pandemic and national regulations, recruitment, and interviews had to be paused in the first half of 2020. JVK, a PhD student and resident oncologist with formal training and experience in qualitative methods, along with a medical student, conducted the interviews separately. Two of the authors, author AL (a general practitioner) and OS (a clinical psychologist), are experienced qualitative researchers and provided guidance concerning prompting questions and contributed to the discussion about data saturation throughout the process.

The interviews were conducted in a private office at the local hospital; due to the ongoing COVID-19 pandemic, all participants declined the offer of being interviewed at home or in a public setting. The duration of the interviews was between 35 and 65 minutes. Memos were written after each interview to better capture its core, as well as the interviewer's initial thoughts, which became the very start of the process of analysis.

### Data Analysis

The interviews were digitally recorded and transcribed verbatim. We analyzed the data inspired by a constructivist grounded theory approach according to Charmaz.^24^ The first step was to read and familiarize ourselves with the text, the second step was initial coding, followed by focused coding and then grouping focused codes into categories. The final step was to examine the relationship between the categories and construct a core-category. The process was abductive, moving back and forth between data and the emerging analysis. For further details about the analysis process, please see Supplementary File.

Group discussion and reflection were essential to the process of defining the research question, recruiting participants, collecting, and analyzing data. Through the keeping of field notes during data collection, the interviewers were made aware of their positioning in relation to the participants and how the data was constructed, which enhanced reflexivity.^25^ During analysis JvK made written memos detailing methodological choices and the interpretation of data that helped to make visible pre-existing knowledge and bias. Constant comparison of the different levels of analysis, in combination with regular discussion prompted by the memos, facilitated the joint decision that theoretical saturation had been obtained.

## Results

When analyzing patients’ experiences of empathy in health care, a recurring narrative was that of wanting to be seen and known, not just regarded as a medical condition. Three categories were formed and inspired us to construct the core category: “I want to be known as a whole human being” (see Table 1).

**Table 1.** Core-Category, Categories and Sub-Categories Based on Patients Experiences of Empathy in Health Care.

Core category	Categories	Subcategories
	I need you to focus on me	Enough time and space to meet properly Showing interest Sharing a common language Non-verbal communication, physical aspects of empathy Feeling left out Feeling like a burden
I want to be known as a whole human being	I know myself and the disease	A shift in knowledge and power balances Being acknowledged as a resource Knowing something beyond the medical agenda about the disease or illness Standardized treatments in non-standardized lives
	I am more than my disease	Being understood in one's context Having to deal with the disease beyond the consultation Feeling vulnerable and exposed Sharing something personal

### I Need you to Focus on Me

The participants described their needs centered around empathy. The need of presence is distinct and described as having enough time to be present in the room and to stay focused on them and their stories. They find it very noticeable when focus shifts during the consultation and that it makes the encounter less empathic. The participants also stress the role of continuity in empathic meetings. Without continuity, they experience the need to repeat themselves many times and feel insignificant.

“They (the doctors) need to show that they want to listen to you. Sitting down in front of you, keeping eye-contact, letting conversations take time. There is no hurry, it is you and me now, let's close the door. They need to show real interest in my story and that I am the most important thing for them at the moment. I think that's a way of showing empathy, let's set all other things a side” **(IP 15)**

The participants perceived active and attentive listening as empathic, and a need for acknowledgement in the form of verbal confirmation. Further, the participants described the need of a shared language, not hindered by accents or, more importantly, cluttered with medical and academic terminology*.* They described non-verbal communication including body language as a way of communicating empathy, in relation to non-verbal communication, the participants described that empathy was of importance in the physical examination. The participants also gave examples of how the lack of empathy rendered negative experiences.

“I had just offered my hand, as to present myself, but the doctor just, no, he didn’t want to, he didn’t want touch me at all. He put on gloves, and then he started to examine my arm. I felt like all I was, was an arm. And then he went on, talking about referring me to the ER. It was so weird. I kind of just stood there, still with my hand, I felt as if I was disgusting.” **(IP 11)**

Further, the participants described situations of feeling like a burden or that they were not evoking enough medical interest from the doctor to render empathy. In relation to this they also described experiences of feeling left out and vulnerable in situations where the doctors did not include them enough in the conversation or just simply neglected their presence.

“The doctor was just waiting for me to finish talking so he could say what he thought. He just wanted me to finish. It wasn't like a collaboration. I think about it in retrospect, it is very easy as a patient to think that I am the one who is troublesome. Next time I won't be so troublesome.” **(IP 11)**

### I Know Myself and the Disease

The participants described that nowadays patients in general have access to greater medical knowledge. Despite this, they experienced that doctors have an idea of knowing more about their patients, than the patients themselves, as well as their diseases. The participants wanted to be included throughout the consultation and be regarded as experts on their own illness. They also wished for a more welcoming attitude towards their medical knowledge regarding their own actual disease, for example regarding new treatments they had read about.

“There is something missing in the consultation sometimes, the patient needs to be regarded as a resource. The interaction becomes so different then, I feel like I am being taken seriously, that my observations matter.” **(IP 11)**

Another problem described by the participants was that healthcare personnel focused too much on standardized treatment programs without considering if, and how, their advice was adaptable to the individual and their daily life. While the participants understood that medical staff have their own agenda, they felt overlooked and less engaged in contact with health care when they weren't regarded as a resource.

“I am my own doctor nowadays; I know myself and I have lived with this disease for more than half my life. And when I think about it, it is important to convey this in education. The fact that the treatment of many diseases is more about taking care of oneself and that health care is more of a support function. The doctors are not the ones dictating how I should live my life” **(IP 15)**

### I am More Than My Disease

The participants described situations where they felt that their doctors only saw their symptoms and diagnosis instead of their more person-oriented needs and wishes. The participants further expressed and elaborated on the need of being seen as a person with context, and for the doctor to consider their personal life and experiences when explaining something, for example, deciding on treatment options. They also described that they sometimes perceived the doctors as only interested in certain parts of their disease or just one condition, even if several other conditions and problems in life affected them.

“You talk about all of your life with the doctor, but then doctor number one just wants to hear part one because it only concerns that disease. And then doctor number two, he just wants to hear about part two, but you have no one who can relate to you as a whole person, a life.” **(IP 17)**

The participants described that when the doctor failed to understand them as a whole human being, they ended up not trusting the doctor and their recommendations regarding treatment. There were also narratives about situations in the consultation where the participants felt vulnerable and exposed, both due to questions of private, sometimes inappropriate, nature and lack of mutual sharing. The participants recounted different strategies for dealing with such situations; sometimes they would share personal stories without being asked to, or they would ask the doctor to share something personal about themself. This was described as an attempt to establish a connection and a way of feeling less vulnerable and exposed—a way to maintain dignity.

“I know nothing about him, about his life and so on. While he knows everything about me and my problem, my physical problem, he knows I think it's so hard because I can’t watch TV. And when you don’t know anything about them (the doctors), how can you relate?” **(IP 17)**

## Discussion and Conclusions

### Discussion

Our analysis resulted in the core category “I want to be known as a whole human being” that contains three categories. The participants’ experiences contribute with a more thorough understanding of how empathy is operationalized in the healthcare setting.

The category “I want you to focus on me” confirms previous findings that empathy is dependent on factors such as communication, verbal as well as non-verbal, and active listening.^4,26^ Further, it specifically stresses the need of a healthcare provider who is present and focused on the patient. Failure to meet these needs leads to distrust,^20^ and the patients refrain from sharing their concerns. We consider these failures dependent on both organizational and individual factors.

The participants in our study seem to be aware of the stressful work environment in health care and are partially willing to accept that it may negatively influence their interaction with healthcare providers. The need for an interaction that is characterized by mutual engagement nevertheless remains evident. For the healthcare provider to be focused and present in the consultation, regardless of organizational prerequisites, we are convinced that engaged curiosity^8^ is crucial. Engaged curiosity creates the possibility to stay present and interested in the patient's story. It is the fundament of a person-centered dialogue between healthcare providers and patients.

We understand the category “I know myself and the disease” as experiences of power imbalances. In our study, power imbalances arise from hierarchies of knowledge, failure to include the patients’ unique experience and from conflicting agendas. These experiences also inform us how a failure to convey empathy hinders shared decision-making; being curious and encouraging enough is a key to the patient's perspective and knowledge.

Should the patient feel excluded and not acknowledged as an expert on their disease and how it affects them, they might lose trust in their healthcare provider and be less willing to communicate their needs. Further, it will decrease their inner motivation for self-care and will to educate themselves about the disease.^27^ For patients with chronic diseases, if this experience persists over time, it may cause general distrust towards health care. Our findings support previous research on patients’ experiences of hierarchies in health care.^28^

Healthcare providers need to acknowledge their patients’ knowledge and experiences of their own illness and disease and try to counteract power imbalances. Our study shows that empathy is fundamental in how the patients perceive the doctors to be curious, inclusive and willing to share the decision-making process. If successful, these efforts will counteract redundant power imbalances, restore patient dignity, and reinforce their self-efficacy leading to increased patient empowerment and enablement.^29^

The category “I am more than my disease” highlights the importance of being understood as a whole human being, with ideas and resources, rather than as a set of symptoms or a diagnosis. Central in their experiences is how the participants connect empathy with being understood as a person within their full context rather than only as a patient in a brief health care interaction. Their narratives exemplify how doctors need to understand the patient's context to establish trust between them, and to tailor recommendations and treatments to their individual needs. This is congruent with earlier research that proves that if the patient feels seen and heard—beyond their symptoms—compliance to treatment as well as patient outcomes can be positively affected.^13-15,30^ Closely related to the contextuality is the need for allowing a personal narrative in the meeting. The participants experiences stress their will to share something personal with their doctor, and that the doctor share something in return, to establish a connection and to relate to each other. We understand the patients need for contextuality and reciprocity as a way of maintaining dignity. This resounds with Post´s^31^ reasoning about empathy being a pathway to maintain patient dignity. We therefore argue that health care providers should dare to be more personal in their interactions with patients.

In sum, we view the shortcomings described by the participants, partly, as the result of a healthcare system that has for a long time focused on symptoms and diseases. The shift towards a more person-centered care has already taken place in theory^9,22^ and in Sweden it is regulated by law.^32^ However, our study shows that this is still difficult in everyday practice. The patients need to be encouraged to be personal and unabridged in their narratives, and to experience mutuality in their relationship with healthcare personnel, is evident in our study and should, in our view, be the main objective for person-centered care.

**Figure 1.**
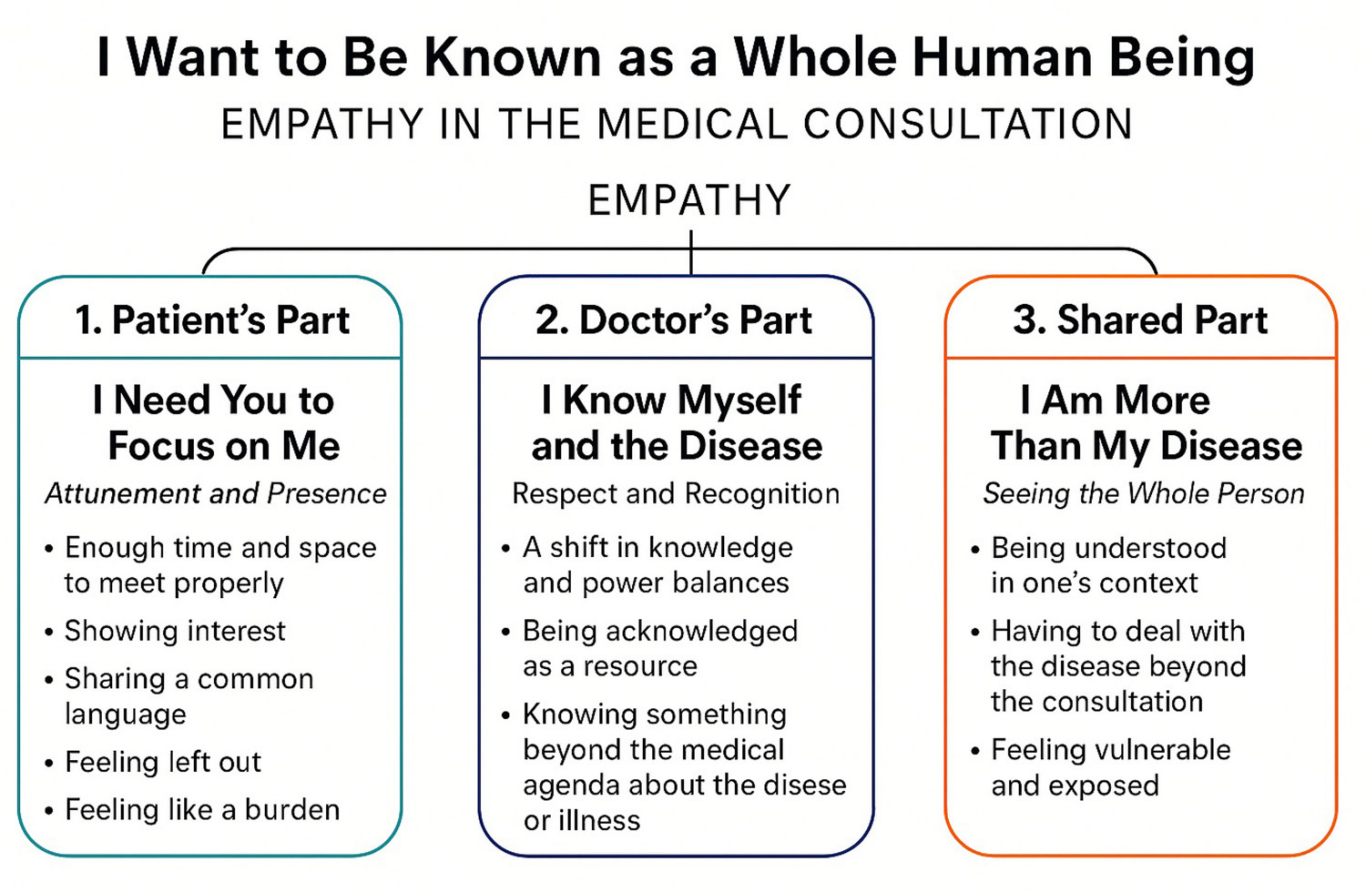
Showing how the findings align within the three part consultation.

The participants’ experiences offer knowledge on how empathy is central to the consultation, further the categories and subcategories highlight how empathy enables a person-centered consultation and the risks of non-empathic consultations. The findings also provide knowledge on critical phases in the consultation process, where empathy is crucial to move the consultation forward in a way that is helpful and maintains the patients trust and dignity. Since the participants’ experiences offered much useful knowledge on how to improve person-centered care, we have constructed a model (Figure 1) that situates the categories in an already well-established consultation framework.^33^ By doing so we hope that clinicians and educators can make use of the findings in a helpful way to improve knowledge of how empathy is necessary for person-centered consultations, enabling existing and future doctors to develop their consultation skills with regards to empathy and person-centeredness.

Further our findings can be useful to inform Stakeholders about the need to further highlight person-centeredness in policy development since there still seems to be a gap between policies and reality. Future research could focus on how to effectively move this knowledge to educational context by for example further involve patients in consultation training, both with students and senior doctors. It could be of interest to investigate through video recorded consultations what factors and processes that patients perceive as person-centered to help healthcare educators and staff to better frame consultation theory and practice in an empathetic and person-centered way.

### Strengths and Limitations

This study is situated in the context of Swedish health care and therefore influenced by contextual aspects of both empathy and the healthcare system. These aspects may have affected the findings in this study, both regarding the general idea of empathy as well as how it was perceived and recalled by the participants, since empathy as well as compassion are culturally tinged phenomena.^5^ The researchers’ different backgrounds, contributed to our awareness of our various preunderstandings; by acknowledging and considering these during data analysis, we enhanced the credibility of our findings. Regarding transferability, our aim is that the descriptions of the researchers’ and the context of the study will make possible for the reader to decide how the findings are transferable to other contexts.^23^

### Conclusions

This study has provided a deeper insight on how empathy can be operationalized in healthcare interactions from a patient perspective. It adds to earlier research on how empathy plays an important part in shared decision-making, patient empowerment and maintaining patient dignity. Our main finding is the patients expressed need to be given the opportunity to be more personal and to experience a mutual sharing with their doctors to not feel vulnerable or left out in the consultation.

We suggest that empathy, partly expressed through engaged curiosity, is the foundation for person-centered care, enabling power to be more equally shared between care provider and patient—which, in turn, increases trust and patient dignity. Most of all empathy enables a genuine encounter between two human beings. Based on this we would like to see that, where the proper organizational structures are in place, students as well as clinicians are encouraged and given the possibility to dare to be more personal in contact with patients.

It would be of interest for future research to examine the “how's and why's” of the failure to apply a person-centered approach (when it in reality ought to have been fully implemented) and how to best facilitate a person-centered approach for students as well as doctors.

## Supplemental Material

sj-docx-1-jpx-10.1177_23743735251369702 - Supplemental material for I Want to be Known as a Whole Human Being: A Qualitative Study About Patients’ Experiences of Empathy in Health Care Supplemental material, sj-docx-1-jpx-10.1177_23743735251369702 for I Want to be Known as a Whole Human Being: A Qualitative Study About Patients’ Experiences of Empathy in Health Care by Johanna von Knorring, Arja Lehti, Kristina Lindvall and Olof Semb in Journal of Patient Experience
