# Supplementary material for: I Want to be Known as a Whole Human Being: A Qualitative Study About Patients’ Experiences of Empathy in Health Care
Source: J Patient Exp. 2025 Aug 21;12:23743735251369702. doi: 10.1177/23743735251369702 (PMC12374116; doi:10.1177/23743735251369702)
Supplement: sj-docx-1-jpx-10.1177_23743735251369702 - Supplemental material for I Want to be Known as a Whole Human Being: A Qualitative Study About Patients’ Experiences of Empathy in Health Care [file sj-docx-1-jpx-10.1177_23743735251369702.docx]

**Supplementary file: Elaboration of the analysis process**

The interviews were digitally recorded and transcribed verbatim. We analyzed the data using a Grounded Theory approach according to Charmaz. First JvK listened to the audio files while reading the transcripts to familiarize with the data. Author n1 took notes on passages that evoked curiosity, as well as how the interviewers interacted with the participants, to reflect upon the preunderstanding at an early stage. Initial coding focused on staying close to the text and was performed by author JvK and author AL, first separately and then together to compare and agree on codes. After reaching a consensus on the initial, inductive codes, author JVK, author AL and author OS continued with grouping of the initial codes and searching for connections between the different code groups while paying attention to not missing any variations or conspicuous experiences. The codes were grouped into categories and subcategories. At this stage author KL, a dietitian and senior researcher specialized in qualitative methods, provided guidance and discussed a subset of the initial and focused codes together with the first author. In addition, author KL contributed with theoretical guidance and new perspective on preliminary sub-categories and categories. Discussing connections and describing the categories was a process of deconstructing and constructing the participants’ experiences. When the final categories, subcategories and core category were agreed upon by all co-authors, author n1 re-read the transcripts to see if any new narratives stood out; no changes were made in the categories after this.
